# Factors associated with age-disparate sexual partnerships among males and females in South Africa: a multinomial analysis of the 2012 national population-based household survey data

**DOI:** 10.1186/s12982-021-00093-5

**Published:** 2021-03-12

**Authors:** Musawenkosi Mabaso, Lungelo Mlangeni, Lehlogonolo Makola, Olanrewaju Oladimeji, Inbarani Naidoo, Yogandra Naidoo, Buyisile Chibi, Khangelani Zuma, Leickness Simbayi

**Affiliations:** 1 Human and Social Capabilities Division, Human Sciences Research Council, Durban, South Africa; 2 Department of Psychology, University of KwaZulu-Natal, 238 Mazisi Kunene Road, Glenwood, Durban, 4041 South Africa; 3 Human and Social Capabilities Division, Human Sciences Research Council, Pretoria, South Africa; 4 Office of the Deputy CEO for Research, Human Sciences Research Council, Cape Town, South Africa; 5 Department of Psychiatry & Mental Health, University of Cape Town, Cape Town, South Africa

**Keywords:** Age-disparate, Sexual partnerships, Multinomial analysis, South Africa

## Abstract

**Background:**

In South Africa, age-disparate to sexual relationships where the age difference between partners is 5 years or greater is an important contributor to the spread of HIV. However, little is known about the predictors of age-disparate sexual relationships. This study investigates factors associated with age-disparate sexual relationships among males and females in South Africa.

**Methods:**

This analysis used the 2012 nationally representative population-based household survey conducted using multi-stage stratified cluster sampling design. Multivariate multinomial stepwise logistic regression models were used to determine factors associated with age-disparate sexual relationships.

**Results:**

Of 15,717 participants, who responded to the question on age-disparate sexual relationships, 62% males versus 58.5% females had partners within 5 years older or younger, 34.7% of males versus 2.7% of females had partners at least 5 years younger and 3.3% of males versus 38.8% of females had partners at least 5 years older. Among both males and females predictors of age-disparate sexual relationships were education, employment, socioeconomic status, locality type, age at sexual debut, condom use at last sexual act and HIV status while race was also an additional predictor for among females. Including unprotected sex and risk of HIV infection among adolescent girls and young women with sexual partners 5 years older their age.

**Conclusions:**

This study suggest that there is a need for reprioritizing the combination of behavioural and structural interventions to address risky sexual behaviours, unprotected sex, poverty, limited education and gender inequitable norms related to age-disparate sexual relationships and HIV.

## Background

In South Africa, age-disparate sexual relationships between partners 5 years or greater is an important contributor to the spread of Human immunodeficiency virus (HIV) [1]. Individuals who age-mix have been shown to be at a greater risk of contracting HIV and are more likely to engage in risky sexual behaviour, such as low condom use and multiple sexual partners [1–5]. This has been attributed to power dynamics in these relationships especially between younger women and older men [1]. Sexual relationships between younger women and older men, in particular, have been widely explored due to heightened risk of HIV amongst young women compared to same aged males and older women [1, 2, 6–9]. Evidence shows that younger women in relationships with older partners are often treated as submissive objects of structural, cultural and economic circumstances that sway their reproductive choices [1–3, 10].

Some researchers, especially in developed countries, have also studied older women in sexual partnerships with young men [11–14]. These types of relationships have also been attributed social and economic influences [11]. Research has also shown that even though there has been an increase in these forms of relationships, there is still a general stigma surrounding these relationships [11–14]. This differs from relationships where there is an older male and a younger female partner which is generally accepted and sometimes even encouraged [1, 2, 12].

Evidence shows that both male and female individuals who have older sexual partners are at a greater risk of HIV infection [15]. In addition, most studies have explored partner age difference as a predictor of HIV infection. However, little is known about the predictors of age difference in sexual relationships. This study investigates factors associated with age-disparate sexual partnerships among males and females in South Africa using the 2012 national population-based household surveys.

## Methods

### Data

Nationally representative survey data from the 2012 South African national household survey based on a multi-stage stratified cluster random household sample were used [9]. The sample comprised 15 visiting points (VPs) or households drawn from 1000 randomly selected enumeration areas (EAs) sampled from 86,000 EAs based on the 2001 census. The selection of EAs was stratified by province and locality type defined as urban formal, urban informal, rural formal (including commercial farms), and rural informal localities (tribal authority) [9].

Persons of all ages living in selected South African households and hostels were eligible to participate in the study. Persons of all ages living in South African households and hostels were eligible to participate. Persons resident in educational institutions, old-age homes, hospitals, correctional facilities and uniformed-service barracks as well as homeless persons were excluded from the survey. A detailed age-appropriate questionnaire was used to collect information related to socio-demographic characteristics, HIV related knowledge, attitudes, and behaviours [9]. The current analysis uses data on youth and adults aged 15 years and older who reported the age of their sexual partner.

### Ethical consideration

The data collection was anonymised to ensure confidentiality. All persons who agreed to participate in the survey were required to provide either written or verbal consent for both the interview and specimen collection. Verbal consent was sort from participants who could not write or read. Centers for Disease Control and Prevention (CDC) granted a waiver of written consent per 45CFR46 for cases where respondents were unable to provide written consent but consented verbally. The survey protocol was approved by the Human Sciences Research Council’s Research Ethics Committee (REC: 5/17/11/10) as well as by the Associate Director of Science of the National Centre for HIV and AIDS, Viral Hepatitis, STD and TB Prevention at the USA’s Centers for Disease Control and Prevention (CDC) in Atlanta.

### Measures

The dependant variable of interest age-disparate sexual relationship was categorised into three levels based on the age of the most recent partner in the last 12 months irrespective of the type of partner, defined as having a sexual partner within 5 years older or younger, sexual partners at least 5 years younger and sexual partners at least 5 years older, making it a multinomial outcome.

Independent variables included socio-demographic characteristics such as age (15 to 24 years vs 25 to 49 years vs 50+ years), race [Black African vs other race groups (i.e., White, Coloured, and Indians/Asians)], marital status (not married vs married), educational level (no education/primary vs secondary vs tertiary), employment status (not employed vs employed), asset based socio-economic status (SES) score made up of a composite measure based on availability essential services and ownership of a range of household assets (high vs middle vs low) and locality type (Urban formal/suburbs vs urban informal/squatter settlements vs rural informal/tribal areas vs rural formal/farms).

Behavioural factors included age at sexual debut (less than 15 years old vs 15 years and older), number of sexual partners in the last 12 months (one partner vs two or more partners), condom use at last sex (no vs yes), alcohol use risk score (abstainers vs low risk vs, high risk vs hazardous risk drinkers) based on the Alcohol Use Disorder Identification Test (AUDIT) scale [16], self-perceived risk of HIV infection (no vs yes), awareness of HIV status (no vs yes) and HIV status (positive vs negative).

### Statistical analysis

Survey data was benchmarked against 2012 mid-year population estimates by age, sex, race, locality type and province to ensure that the sample estimates were generalisable to the respective populations of South Africa. Frequency distribution and percentages were used to describe the socio-demographic and behavioural characteristics of the study sample. Pearson chi-square test was used to test for differences between categorical variables. Multivariate multinomial logistic regression models using backward stepwise selection method were fitted to determine factors independently associated with age-disparate relationships stratified by sex and using sexual partner within 5 years older or younger as reference category. Age-disparate patterns were also assessed in age-stratified models for 15–25-year olds, 25–49 year olds, and those 50 years and older males and females. Relative risk ratio (RRR) computed as the exponentiated coefficient from -mlogit- with 95% confidence intervals (CI) was used as a measure the effect of each variable on age-disparate sexual relationships. The “svy” command was used to introduce weights which take into account the complex multilevel survey design. All statistical analysis was significant at a p-value ≤ 0.05. STATA Statistical Software Release 13.0 was used to conduct analysis of the data (Stata Corporation, College Station, Texas, USA).

## Results

### Characteristics of the study sample

A sub-sample of 15,717 participants was used in the analysis, 51.6% 48.4 (n = 7380) males and 48.4% females (n = 8337). The majority of the participants were aged 25 to 29 years, Black African, not married, had secondary level education, were unemployed, and were from low (SES) households in urban formal areas (Table 1).

**Table 1:** Socio-demographic characteristics of the study sample (n = 15,717)

	All		Males		Females	
	Total^a^	%	n	%	n	%
Age (years)						
15 to 24	3329	21.7	1559	20.4	1770	23.0
25 to 49	9096	62.9	4010	61.8	5086	64.2
50+	3292	15.4	1811	17.8	1481	12.8
Race groups						
Black African	8989	77.6	4112	77.1	4877	78.1
Others	6717	22.4	3260	22.9	3457	21.9
Marital status						
Not married	8091	59.1	3860	60.0	4231	58.1
Married	7441	40.9	3422	40.0	4019	41.9
Education level						
No education/primary	2172	15.3	1051	16.0	1121	14.6
Secondary	10,059	71.4	4637	70.4	5422	72.5
Tertiary	1646	13.2	815	13.5	831	12.9
Employment status						
Unemployed	7282	52.3	2452	42.2	4830	63.0
Employed	7453	47.7	4361	57.8	3092	37.0
Asset based SES						
Low SES	5474	43.4	2551	42.5	2923	44.3
Middle SES	5019	33.6	2275	33.0	2744	34.2
High SES	5006	23	2458	24.5	2548	21.4
Locality type						
Urban formal	9375	53.4	4483	53.8	4892	52.9
Urban informal	1753	8.7	810	9.0	943	8.4
Rural informal	2930	31.8	1246	30.3	1684	33.5
Rural formal	1659	6.1	841	6.9	818	5.2

*SES* Socio-Economic Status

^a^Subtotals do not add up to the overall Total (n) due to non-response and/or missing data

Most participants reported age of sexual debut at the age of 15 years and older, had one sexual partner, were abstainers, self-perceived themselves as being at risk of HIV, were not aware of their HIV status and were mainly HIV negative (Table 2).

**Table 2:** Behavioural characteristics of the study sample (n = 15,717)

	All		Males		Females	
	Total^a^	%	n	%	n	%
Age at sexual debut						
Younger than 15 years	860	7.2	588	10.4	272	3.9
15 years and older	13,764	92.8	6143	89.6	7621	96.1
Sexual partners in the last						
1 partner	14,121	87.5	6170	80.0	7951	95.5
2+ partner	1453	12.5	1134	20.0	319	4.5
Condom use last sex act						
No	10,650	63.8	4776	61.4	5874	66.5
Yes	4687	36.2	2402	38.6	2285	33.5
Alcohol use risk score^b^						
Abstainers	9246	65.6	3250	50.3	5996	81.8
Low risk drinkers (1–7)	3864	28.1	2585	39.5	1279	16.1
High risk drinkers (8–19)	768	5.9	616	9.7	152	1.9
Hazardous drinkers (20+)	42	0.3	36	0.5	6	0.1
Risk of HIV infection						
No	3678	29.1	1479	25.9	2199	32.5
Yes	11,882	70.9	5833	74.1	6049	67.5
HIV status						
Negative	10,311	80.2	4919	84.3	5392	76.2
Positive	1827	19.8	618	15.7	1209	23.8
Awarenes of HIV status?						
No	8198	51.0	4246	57.8	3952	43.7
Yes	7250	49.0	3011	42.2	4239	56.3

*SES* Socio-Economic Status

^a^Subtotals do not add up to the overall Total (n) due to non-response and/or missing data

^b^Alcohol risk score based on a questionnaire for Alcohol Use Disorder Identification Test (AUDIT)

### Age-disparate sexual relationships and sample characteristics

**Fig. 1:**
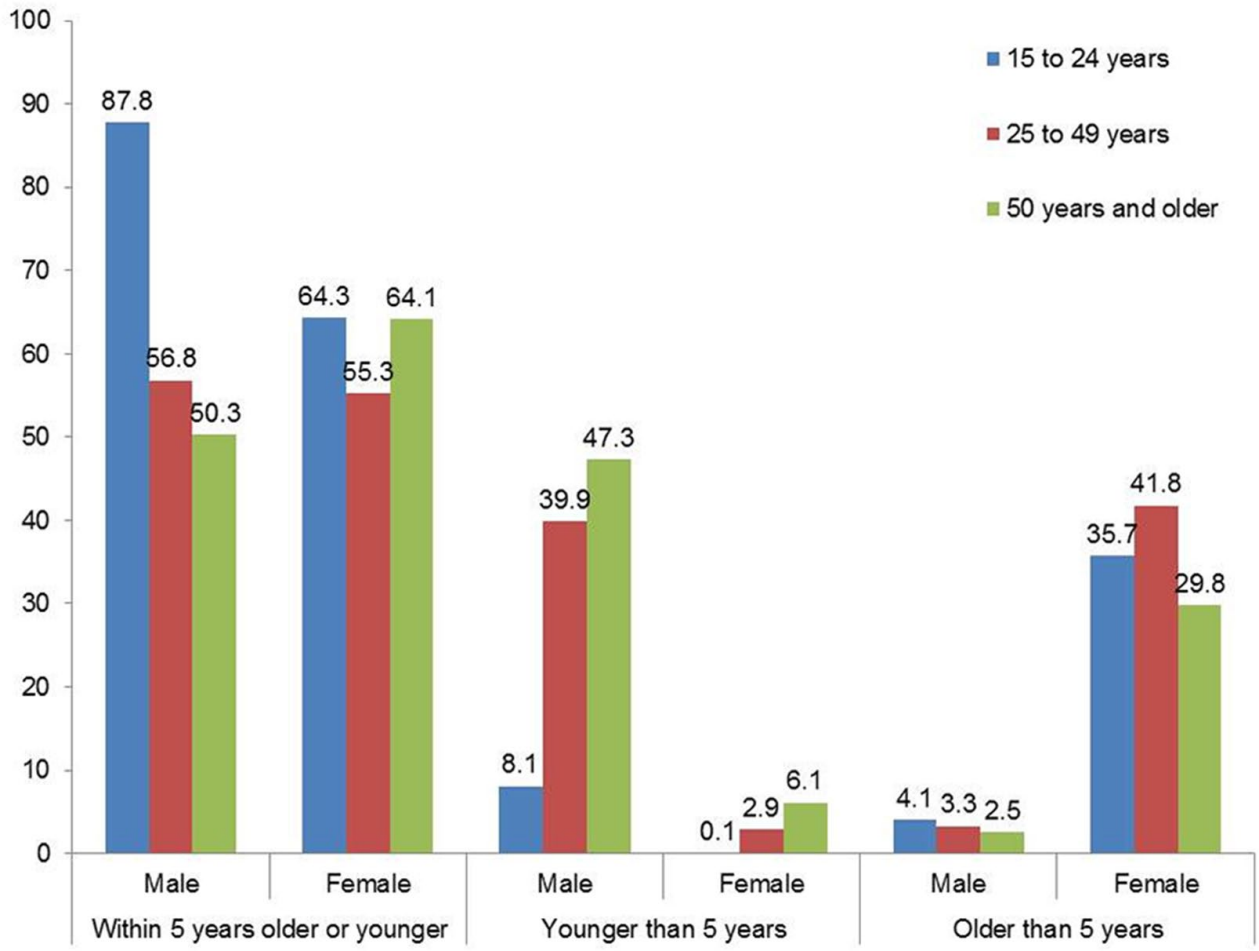
Distribution of age-disparate sexual relationships by gender and sex

Out of the sub-sample 15,717 participants that responded to the question on age-disparate, 62% (n = 7380) males versus 58.5% (8337) females had partners within 5 years older or younger, 34.7% males versus 2.7% females had partners at least 5 years younger and 3.3% males versus 38.8% females had partners at least 5 years older. Figure 1 shows that a higher proportion of males aged 15–24 years had sexual partners within 5 years older or younger compared to females. In addition, a higher proportion of males 25–49 years and those 50 years and older had sexual partners by at least 5 years compared to females while a higher proportion of females aged 15–24 years, 25–49 years and those 50 years and older had sexual partners older by at least 5 years compared to males.

There was a statistically significant difference in age-disparate sexual relationships by race, education level, household SES, and locality type among males (Table 3). Sexual partners within 5 years of age were reported more among other race groups than Black Africans and the opposite was true for sexual partners 5 years younger. Furthermore, sexual partners within 5 years of age were reported more among males with tertiary education than those with no schooling and/or primary level education and vice versa for sexual partners 5 years younger. Sexual partners within 5 years of age were also commonly reported among those from high SES household than low SES household and inversely sexual partners 5 years younger were commonly reported among those from low than high SES households. In addition, sexual partners within 5 years of age were reported more among those from urban formal and rural formal areas than those from urban informal and rural informal areas and the inverse was true for sexual partners 5 years younger.

**Table 3:** Age disparate sexual relationships and socio-demographic characteristics of the study participants by sex

Variables	Males								Females							
	n	Within 5 years older or younger		Younger than 5 years		Older than 5 years			n	Within 5 years		Younger than 5 years		Older than 5 years		p-value
		%	95% CI	%	95% CI	%	95% CI	p-value		%	95% CI	%	95% CI	%	95% CI	
Race groups																
Black African	4112	59.5	57.0–61.9	37.5	35.1–39.9	3.0	2.3–4.0	< 0.001	4877	56.0	53.9–58.1	2.1	1.5–3.0	41.8	39.7–44.0	< 0.001
Others	3260	70.3	67.0–73.4	25.3	22.3–28.6	4.3	3.3–5.7		3457	67.4	64.6–70.0	4.5	3.5–5.8	28.1	25.7–30.7	
Marital status																
Not married	3860	62.1	59.5–64.6	34.2	31.7–36.8	3.7	2.8–4.9	0.44	4231	60.4	58.0–62.7	3.2	2.4–4.3	36.4	34.1–38.7	< 0.001
Married	3422	61.3	58.0–64.5	35.8	32.8–39.0	2.9	2.1–4.0		4019	55.9	53.4–58.4	1.8	1.4–2.4	42.3	39.8–44.9	
Education level																
No education/Primary	1051	48.9	44.3–53.5	48.4	43.7–53.1	2.7	1.7–4.3	< 0.001	1121	52.5	48.3–56.5	2.9	2.0–4.3	44.6	40.7–48.7	0.021
Secondary	4637	64.7	62.2–67.2	32.0	29.7–34.4	3.3	2.5–4.4		5422	60.3	58.0–62.4	2.3	1.8–3.0	37.4	35.3–39.6	
Tertiary	815	68.3	62.4–73.7	29.3	24.1–35.2	2.4	1.0–5.3		831	61.8	55.5–67.7	4.7	2.1–10.4	33.5	27.8–39.7	
Employment status																
Unemployed	2452	62.8	59.6–65.8	33.6	30.5–36.8	3.7	2.6–5.2	0.746	4830	57.2	55.1–59.1	1.9	1.5–2.4	41.0	39.0–43.0	0.001
Employed	4361	61.8	59.3–64.3	34.9	32.3–37.5	3.3	2.5–4.3		3092	60.2	56.7–63.6	3.8	2.6–5.5	36.0	32.7–39.5	
Asset based SES																
Low SES	2551	56.8	53.8–59.7	41.0	38.1–44.0	2.2	1.6–3.1	< 0.001	2923	54.6	52.1–57.0	2.5	1.6–3.9	42.9	40.4–45.5	< 0.001
Middle SES	2275	61.5	58.2–64.7	34.3	31.0–37.8	4.1	2.9–6.0		2744	59	55.6–62.3	2.8	2.0–3.8	38.2	35.0–41.6	
High SES	2458	71.8	68.3–75.0	24.9	21.9–28.2	3.3	2.3–4.8		2548	66	62.6–69.4	2.8	2.0–4.0	31.1	27.9–34.5	
Locality type																
Urban formal	4483	65.4	62.6–68.0	30.5	27.9–33.2	4.1	3.2–5.3	< 0.001	4892	61.4	58.7–64.0	3.1	2.5–4.0	35.4	32.9–38.0	< 0.001
Urban informal	810	56.6	51.5–61.6	41.8	37.0–46.9	1.6	0.8–2.9		943	56.1	51.6–60.6	1.8	1.0–3.2	42.1	37.7–46.6	
Rural informal	1246	55.5	51.8–59.2	42.0	38.4–45.6	2.5	1.4–4.4		1684	53.5	50.6–56.3	2.0	1.0–3.9	44.5	41.4–47.8	
Rural formal	841	70.7	64.2–76.4	26.0	21.0–31.8	3.3	1.9–5.6		818	65.4	59.9–70.4	3.3	2.0–5.6	31.3	26.7–36.3	

There was also a statistically significant difference in age-disparate sexual relationships by race, marital status, education level, employment status, household SES, and locality type among females (Table 3). Sexual partners 5 years younger were reported more among other race groups than Black African, and the opposite was true for sexual partners 5 years older. Sexual partners within 5 years of age were also reported more among those not married and sexual partners 5 years older among those married. In addition, sexual partners within 5 years of age were commonly reported among females with tertiary education and secondary level education than those with no schooling and /or primary level education and vice versa for sexual partners 5 years older. Sexual partners within 5 years of age were reported more among those employed than the unemployed and sexual partners 5 years older among those unemployed than the employed. Furthermore, sexual partners within 5 years of age were commonly reported among females from high SES household than medium SES and low SES household and the inverse was true for sexual partners 5 years older. Sexual partners within 5 years of age were also commonly reported among those from urban formal and rural formal areas than those from urban informal and rural informal areas and the opposite was true for sexual partners 5 years older.

Table 4 shows that among males there was a statistically significant difference in age-disparate sexual relationships by age at sexual debut, alcohol use, self-perceived risk of HIV infection, and HIV status. race, education level, household SES, and locality type. Sexual partners within 5 years of age were reported more among males whose age of sexual debut was younger than 15 years and the inverse was true for sexual partners 5 years younger. In addition, sexual partners within 5 years of age were commonly reported among those who were low alcohol risk users, and sexual partners 5 years younger among hazardous alcohol users. Sexual partners within 5 years were also reported more among those who perceived themselves as being at risk of HIV infection and sexual partners 5 years younger among those who did not perceived themselves as being at risk of HIV infection. Sexual partners within 5 years were also commonly reported among those who tested negative for HIV and the opposite was true for sexual partners 5 years younger.

**Table 4:** Age disparate sexual relationships and behavioural characteristics of study participants by sex

Variables	Males								Females							
	n	Within 5 years		Younger than 5 years		Older than 5 years			n	Within 5 years		Younger than 5 years		Older than 5 years		p-value
		%	95% CI	%	95% CI	%	95% CI	p-value		%	95% CI	%	95% CI	%	95% CI	
Age at sexual debut																
Younger than 15	588	72.0	66.0–77.4	26.2	20.9–32.4	1.7	1.0–3.0	0.001	272	52.1	44.5–59.6	4.4	1.9–9.9	43.5	36.1–51.1	0.164
15 and older	6143	61.0	58.7–63.3	35.5	33.2–37.8	3.5	2.7–4.4		7621	58.9	57.1–60.6	2.6	2.0–3.3	38.6	36.8–40.4	
Sexual partners in the last 12 months																
One partner	1134	62.9	58.6–66.9	33.9	29.6–38.4	3.3	2.0–5.3	0.864	319	62.0	53.4–69.9	2.9	1.1–7.5	35.0	27.5–43.4	0.639
Two or more partners	6170	61.6	59.4–63.7	35.1	32.9–37.2	3.4	2.6–4.3		7951	58.3	56.5–60.1	2.6	2.1–3.3	39.0	37.2–40.8	
Condom use last sex act																
No	4776	60.8	58.3–63.4	35.2	32.7–37.8	3.9	3.1–5.0	0.136	5874	57.4	55.0–59.8	3.0	2.3–4.0	39.6	37.2–41.9	0.077
Yes	2402	63.3	59.9–66.5	34.3	31.0–37.7	2.4	1.5–3.9		2285	60.1	57.5–62.7	1.9	1.3–2.8	37.9	35.3–40.6	
Alcohol use risk score^a^																
Abstainers	3250	59.1	56.4–61.7	37.4	34.8–40.0	3.5	2.5–5.0	0.034	5996	57.1	55.0–59.1	2.3	1.7–3.1	40.6	38.5–42.8	< 0.001
Low risk drinkers (1–7)	2585	64.8	61.8–67.7	32.1	29.2–35.2	3.0	2.2–4.2		1279	65.3	61.0–69.3	4.3	3.0–6.1	30.4	26.7–34.5	
High risk drinkers (8–19)	616	59.1	52.7–65.2	36.0	30.2–42.3	4.9	2.7–8.8		152	57.4	42.1–71.4	2.9	0.9–9.0	39.7	26.3–54.8	
Hazardous drinkers (20+)	36	42.5	23.2–64.5	45.1	24.7–67.2	12.5	2.1–48.0		6	66.8	23.6–92.9	9.2	1.1–47.6	23.9	3.4–73.9	
Self-perceived risk of HIV infection																
No	1479	57.0	52.8–61.1	38.6	34.8–42.6	4.4	2.8–6.8	0.015	2199	52.9	49.8–56.0	3.3	2.3–4.7	43.8	40.6–47.0	< 0.001
Yes	5833	63.6	61.4–65.8	33.4	31.3–35.6	3.0	2.3–3.8		6049	61.4	59.2–63.4	2.4	1.9–3.0	36.3	34.3–38.3	
Awareness of HIV status?																
No	4246	63.6	61.1–66.1	33.2	30.9–35.7	3.2	2.4–4.1	0.156	3952	60.0	57.3–62.5	2.1	1.6–2.8	37.9	35.3–40.6	0.099
Yes	3011	59.6	56.4–62.8	36.7	33.6–39.9	3.7	2.5–5.3		4239	57.5	55.3–59.7	3.1	2.3–4.2	39.4	37.2–41.6	
HIV status																
Negative	4919	62.7	60.3–65.0	33.9	31.6–36.2	3.4	2.6–4.5	0.005	5392	59.4	56.8–61.9	2.4	1.9–3.0	38.3	35.9–40.7	< 0.001
Positive	618	53.0	47.0–58.8	42.9	37.3–48.7	4.1	2.4–7.0		1209	51.5	48.0–55.0	4.1	2.5–6.6	44.4	40.4–48.4	

^a^Alcohol risk score based on a questionnaire for Alcohol Use Disorder Identification Test (AUDIT)

Table 4 also shows that among females there was a statistically significant difference in age-disparate sexual relationships by alcohol use, self-perceived risk of HIV infection, and HIV status. Sexual partners within 5 years of age were commonly reported among those who were low alcohol risk users, and sexual partners 5 years older among hazardous alcohol users. In addition, sexual partners within 5 years were reported more among those who perceived themselves as being at risk of HIV infection and the opposite was true for sexual partners 5 years older. Sexual partners within 5 years were also commonly reported among those who tested negative for HIV and the opposite was true for sexual partners 5 years older.

### Multivariate analysis of factors associated with of age-disparate sexual relationships

#### Female model

**Fig. 2:**
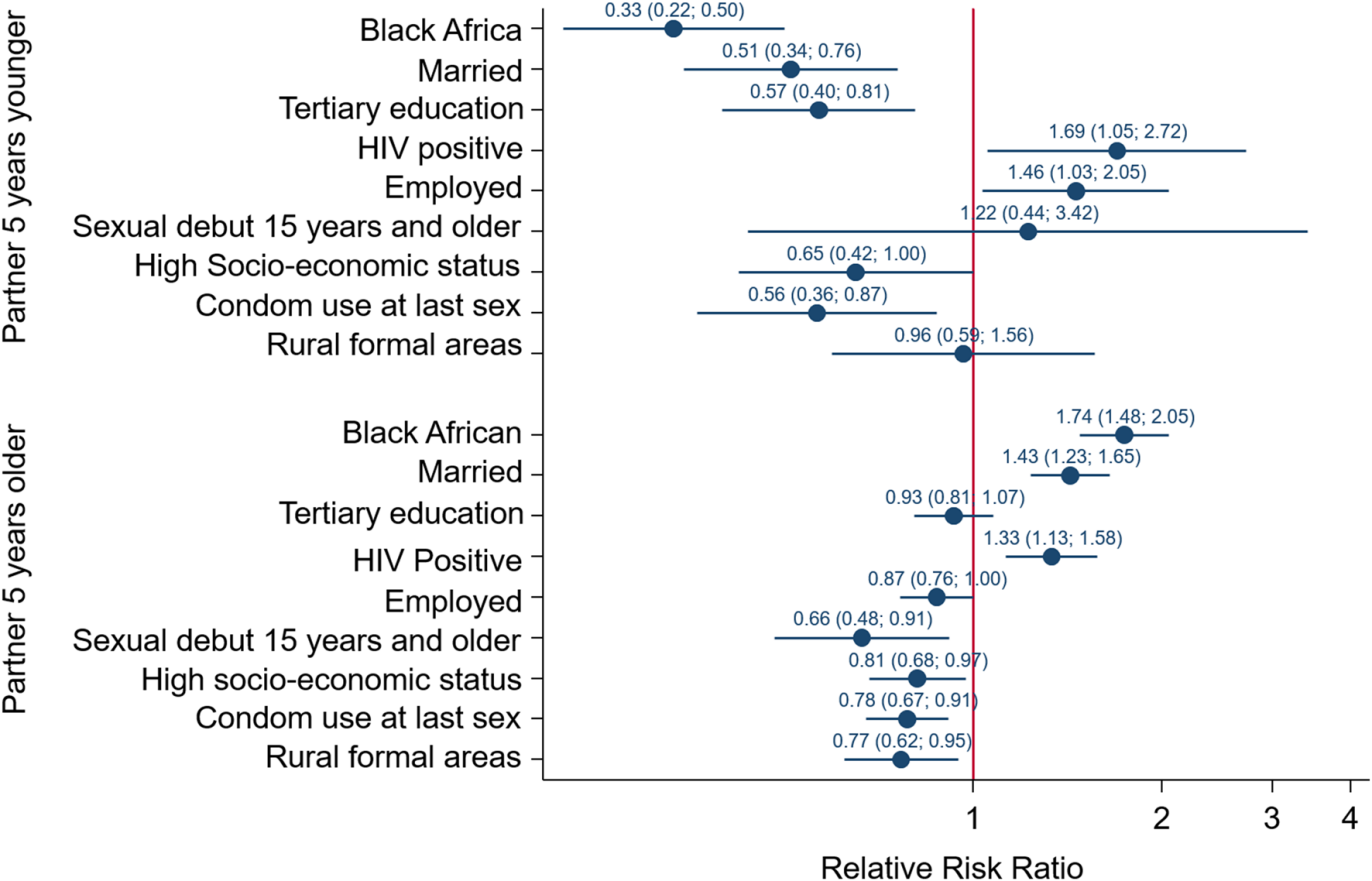
Coefficient plots of multivariate multinomial stepwise regression model of factors associated with age-disparate sexual relationships in females

Figure 2 shows that relative to females who reported having a sexual partner within 5 years of their age, females who were Black African, (RRR = 0.33, 95% CI 0.22–0.50) married RRR = 0.51, 95% CI 0.34–0.76), had tertiary level education (RRR = 0.57, 95% CI 0.40–0.81), and lived in high SES household (RRR = 0.65, 95% CI 0.42–1.00) and used a condom at last sex (RRR = 0.56, 95% CI 0.36–0.87) were significantly less likely to have a sexual partner 5 years younger than those of other races, unmarried, with no schooling or only primary level education and living in low SES households. Females who were employed (RRR = 1.46, 95% CI 1.03–2.05) and HIV positive (RRR = 1.69, 95% CI 1.05–2.72) were significantly more likely to have a sexual partner 5 years younger than unemployed and HIV negative females.

Furthermore, females who had sexual debut at 15 years and older [RRR = 0.66 (95% CI 0.48–0.91), p = 0.012], employed [OR = 0.87 (95% CI 0.76–1.00), p = 0.047], living in high SES household [RRR = 0.81 (95% CI 0.68–0.97), p = 0.022], living in formal rural areas [RRR = 0.77 (95% CI 0.62–0.95), p = 0.013] and used a condom at last sex [RRR = 0.78 (95% CI 0.67–0.91), p = 0.002] were significantly less likely to have a sexual partner 5 years older compared to those who had sexual debut at less than 15 years old, unemployed, in urban areas and did not use a condom at last sex. In addition, female who were Black African [RRR = 1.74 (95% CI 1.48–2.05), p < 0.001], married [RRR = 1.43 (95% CI 1.23–1.65), p < 0.001] and HIV positive [RRR = 1.33 (95% CI 1.13–1.58), p = 0.001] were significantly more likely to have a sexual partner 5 years older than those of other race groups.

#### Female models stratified by age

**Fig. 3:**
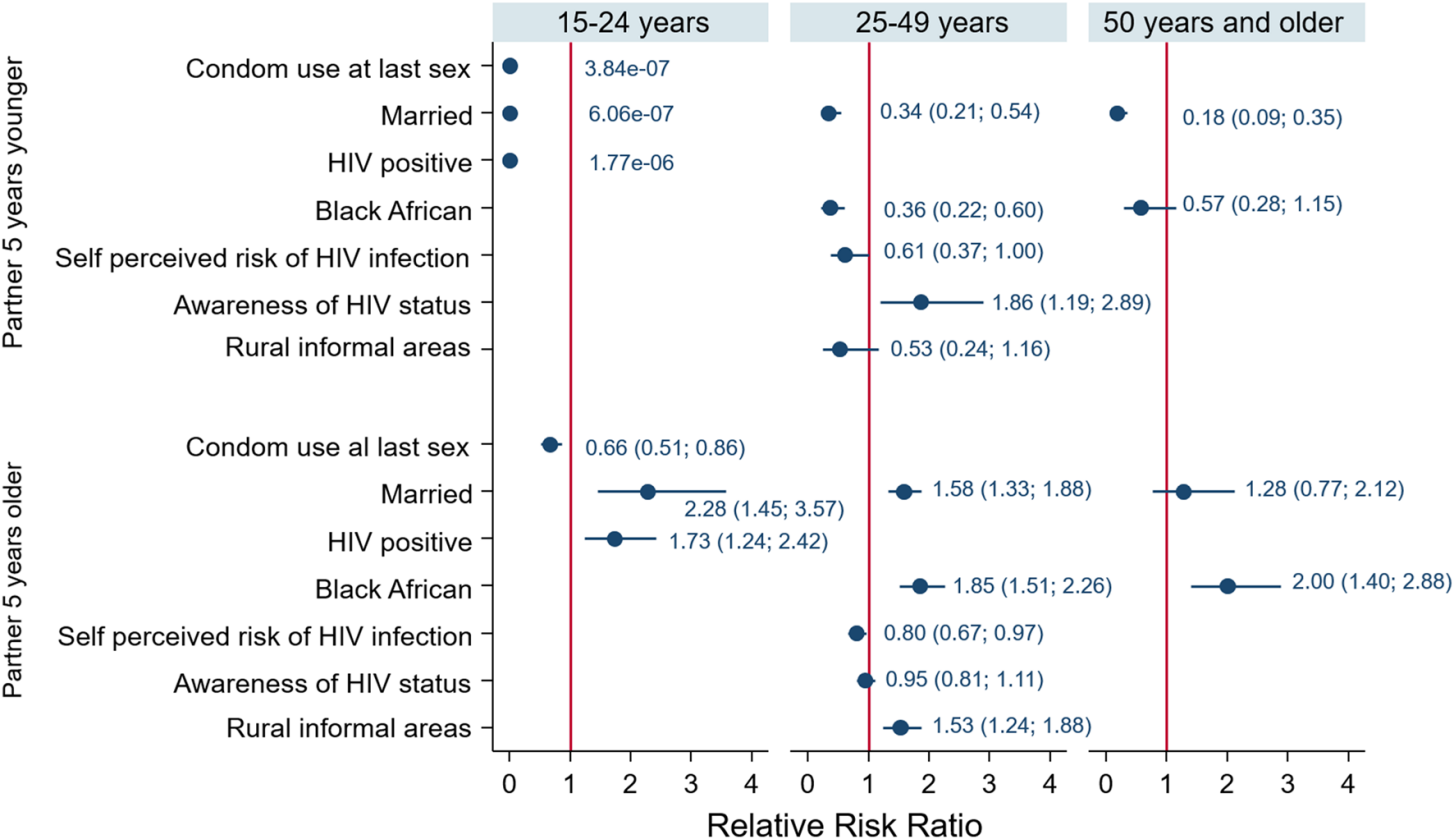
Coefficient plots of multivariate multinomial stepwise regression model of factors associated with age-disparate sexual relationships stratified by age of female participants

The female male model of those 15–24 years (Fig. 3) shows that relative to females who have sexual a partner within 5 years of their age, females who used a condom at last sex [RRR = 0.66 (95% CI 0.51–0.86), p < 0.001], married (RRR = 2.28, 95% CI 1.45–3.57) and HIV positive (RRR = 1.73, 95% CI 1.24–2.42) were significantly less likely to have a sexual partner 5 years older compared to those who used a condom at last sex, unmarried and HIV negative.

The female model of those 25–49 years (Fig. 3) shows that relative to females who have a sexual partner within 5 years of their age, females who were Black Africans (RRR = 0.36, 95% CI 0.22–0.60) married (RRR = 0.34, 95% CI 0.21–0.54), perceive themselves as being at risk of HIV (RRR = 0.61, 95% CI 0.37–1.00), aware of their HIV status (RRR = 1.86 (95% CI 1.19–2.89) were significantly less likely to have a sexual partner 5 years younger compared to those of other race groups, unmarried, and who did perceive themselves as being at risk of HIV and not aware of their HIV status. Females who were Black Africans (RRR = 1.85, 95% CI 1.51–2.26), married (RRR = 1.58, 95% CI 11.33–1.88), living in rural informal areas (RRR = 1.53, 95% CI 1.24–1.88) were significantly more likely to have a sexual partner older than 5 years compared to those of other race groups, unmarried, and living in urban areas. In addition, females who perceive themselves as being at risk of HIV (RRR = 0.80, 95% CI 0.67–0.97) were significantly less likely to have a sexual partner older than 5 years compared to those who did not perceive themselves as being at risk of HIV.

The female model of those 50 years and older (Fig. 3) shows that relative to females who have a sexual partner within 5 years of their age, females who were married (RRR = 0.18, 95% CI 0.09–0.35) were significantly less likely have sexual partners 5 years younger than unmarried females. Black African female (RRR = 2.00, 95% CI 1.40–2.88) were significantly less likely to have sexual partners older than 5 years were than females of other race groups.

#### Male model

**Fig. 4:**
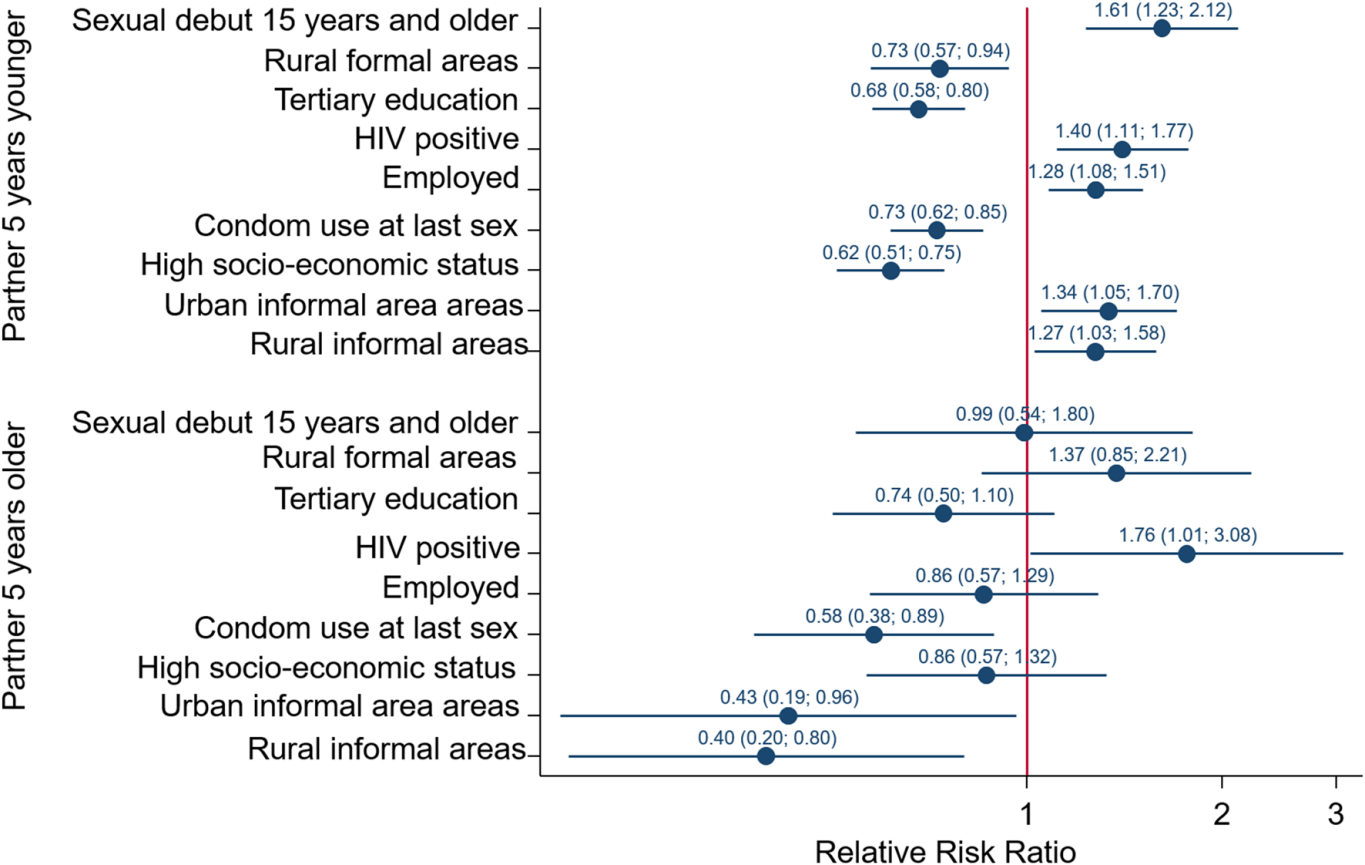
Coefficient plots of multivariate multinomial stepwise regression model of factors associated with age-disparate sexual relationships in males

Figure 4 shows that relative to males who reported having sexual a partner within 5 years of their age, males who had tertiary education (RRR = 0.68, 95% CI 0.58–0.80), in high SES households (RRR = 0.62, 95% CI 0.51–0.75), in rural formal areas (RRR = 0.73, 95% CI 0.57–0.94) and used a condom at last sex (RRR = 0.73, 95% CI 0.62–0.85) were significantly less likely to have a sexual partner 5 years younger compared to those with no schooling and /or primary level education, living in low SES households, living in urban areas and who did not use a condom at last sex. In addition, males who had sexual debut at 15 years and older (RRR = 1.61, 95% CI 1.23–2.12), employed (RRR = 1.28, 95% CI 1.08–1.51), living in informal urban areas [RRR = 1.34 (95% CI 1.05–1.70), p = 0.018], and informal rural areas (RRR = 127, 95% CI 1.03–1.58), and HIV positive (RRR = 1.40, 95% CI 1.11–1.77), p = 0.005] were significantly more likely to have a sexual partner 5 years younger compared to those who had sexual debut younger than 15 years old, unemployed, living in urban areas, and HIV negative. Furthermore, relative to males who reported having sexual partners within 5 years of their age, males living in informal urban areas (RRR = 0.43, 95% CI 0.19–0.96), and informal rural areas (RRR = 0.40, 95% CI 0.20–0.80), were significantly less likely to have a sexual partner older than 5 years than those living in urban areas. HIV positive males (RRR = 1.76, 95% CI 1.01–3.08) were significantly more likely to have a sexual partner older than 5 years compared to HIV negative males.

#### Male models stratified by age

**Fig. 5:**
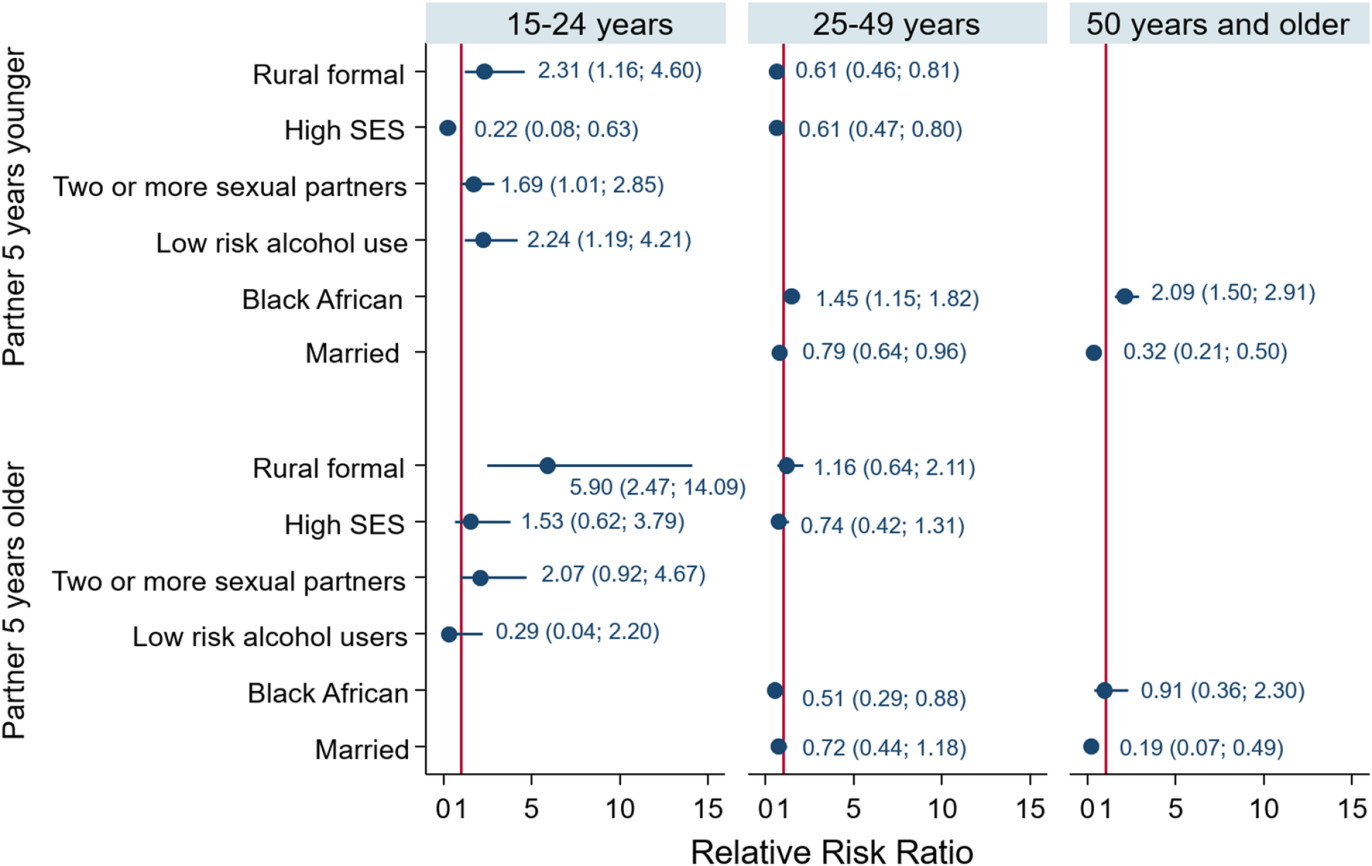
Coefficient plots of multivariate multinomial stepwise regression model of factors associated with age-disparate sexual relationships stratified by age of male participants

The male model of those 15–24 years (Fig. 5) shows that relative to males who have a sexual partner within 5 years of their age, males with two or more sexual partners (RRR = 1.69, 95% CI 1.01–2.85), low risk drinkers (RRR = 2.24, 95% CI 1.19–4.21) and living in rural formal areas (RRR = 2.31, 95% CI 1.16–4.60) and high SES households (RRR = 0.22, 95% CI 0.08–0.63) were significantly less likely to have sexual partners 5 years younger compared to those with one sexual partner, abstainers from alcohol, living in urban areas and living in low SES households. Males living in rural formal areas (RRR = 5.90, 95% CI 2.47–14.09) were significantly more likely to have a sexual partner older than 5 years compared to those in urban areas.

The male model of those 25–49 years (Fig. 5) shows that relative to males who have sexual partners within 5 years of their age, males who were married (RRR = 0.79, 95% CI 0.64–0.96), living in high SES household, (RRR = 0.61, 95% CI 0.47–0.80), living in formal rural area (RRR = 0.61, (95% CI 0.46–0.81) were significantly less likely to have sexual partners 5 years younger compared to those who were unmarried, living in low SES households and living in urban informal areas. Black Africans males (RRR = 1.45, 95% CI 1.15–1.82), p = 0.002] were significantly more likely to a sexual partner 5 years younger than males of other race groups. They were significantly less likely [RRR = 0.51 (95% CI 0.29–0.88), p = 0.015] to have sexual partners older than 5 years compared to males of other race groups.

The male model of those 50 years and older (Fig. 3) shows that relative to males who have sexual partners within 5 years of their age, males who were married [RRR = 0.32 (95% CI 0.21–0.50), p < 0.0001], and Black African [RRR = 2.09 (95% CI 1.50–2.91), p < 0.0001] were significantly less likely to have a sexual partner 5 years younger compared to unmarried males and those of other race groups. Married males [RRR = 0.19 (95% CI 0.07–0.49), p = 0.001] were less likely to have a sexual partner older than 5 years compared to unmarried males.

## Discussion

The findings illustrate that the most common age-disparate sexual partnerships are those between young women and older men. This is in line with observations from other studies [1, 2, 6, 8]. The findings also reaffirm that those engaged in an age-disparate relationship of any kind are more likely to be HIV positive. Other studies have shown that women who have sex with older men are at a greater risk of contracting HIV [1, 2, 8] while some have found that age-disparate sexual relationships do not influence HIV prevalence [6, 7]. The current findings also point to the increased risk to HIV infection amongst men and women who have sexual partners who are either younger or older compared to those with same age sexual partners. These finding suggest an urgent need for prevention interventions to mitigate drivers of age disparate sexual relationships in order to reduce HIV transmission in the country.

The current findings show that amongst females, race, marital status, education, socioeconomic status, locality type, sexual debut, and condom use at last sex were also associated with age-disparate sexual relationships. The historical systematic oppression of black people in South Africa has resulted in socioeconomic disparities that remain to this day, particularly for women [17, 18]. This means that black women in South Africa are more likely to be in socioeconomic difficulties and therefore more likely to look to older and more financially stable partners for socioeconomic stability.

These observations are consistent with the suggestion that women of a lower socio-economic status engage in relationships with older men as a way of mitigating their socioeconomic limitations, as older men are seen as more economically stable [1–3, 10]. The observed urban rural urban divide in age-disparate sexual relationships suggests that normative limitations regarding the relationships between older men and younger women present in rural areas break down in urban areas and allow for the greater presence of these relationships [1]. In addition, women who have early sexual debuts are more likely to have grown up in poverty stricken households suggesting that they may engage in partnerships with older men in part for financial sustenance [1].

The age-stratified female models showed that young women (15–25 years) were less likely to have same age sexual partners. Those with partners 5 years older were less likely to use condom and more likely to be married and HIV positive. These findings support the notion that most young women who engage in relationships with older men do so because they are seeking stable partnerships and even marriage. Older men are seen as more serious and better able to take care of the women and their offspring [1, 4, 19]. As observed in the current study, younger females in such relationships have less power to negotiate the safe sexual encounters [8, 19]. Young women involved in such relationships are at greater risk of HIV infection due to unprotected sex with such sexual partners [8].

The results also showed that older women (25–49 years) with sexual partners five younger than their age were less likely to be Black African and married and did not perceived themselves as not being at risk of HIV infection. While those with sexual partners 5 years older than their age resided mainly in rural areas were more likely to be Africa and married and did not perceive themselves as being at risk of HIV.

In the male model age-disparate was also associated with education, employment, socioeconomic status, locality type, sexual debut and condom use at last sex. Similar to the female models these findings reflect economically driven gender inequalities in age mixed sexual relationships, which are linked to male partner characteristics. For example, higher education is associated with employment and higher income that allows for more disposable income for social activities. Age stratified model models showed that young men (15–25 years) with sexual partners younger than them were more likely to have multiple sexual partners and to leave in low SES household in urban informal areas. While older man (15–49) with sexual partners younger than them were more likely to be Black African, leave in high SES household and less likely to be married.

The “sugar daddy” phenomenon where younger girls offer sexual services to older men in exchange for gifts is facilitated by the older men’s higher socio-economic status [19]. Impoverished and vulnerable young women in informal settlements are ‘pulled’ into such relationships in the hope of a better life [1]. In such instances, young women are expected to be obedient and respectful toward older men, which undermine their ability to resist older men’s advances and negotiate condom use. As reflected in the females model the fact that males who had younger sexual partners were less likely to have used condom at last sex can be attributed to the gender power disparities between them and their younger partners [19]. These older men, due to their age and economic sway have the prerogative regarding sexual practices [2, 19].

These findings accentuate the importance of a combination of behavioural and structural interventions to reduce factors that mediate between age-disparate relationships and HIV transmission premised. Such interventions should be premised on the understanding that individual behaviours are not randomly distributed within a population but are instead perpetuated by the interaction of the individuals with these factors [20]. The current findings suggest that factors that mediate between age-disparate relationships and HIV transmission in include among others poverty, limited education and gender inequitable norms.

### Limitations

The study used a cross-sectional study design and therefore cannot infer causality but is limited only to establishing association between age-disparate relationships and potential predictors. The data were collected based on self-report that might be subject to social desirability and recall bias. The survey was not designed to sample marginalised, hidden and hard to reach populations and is therefore subject to selection bias. Furthermore, both unit and item non-response results in the reduction in sample size and statistical power to detect the deference or associations. However, sampling weights which accounts for the complex survey design were adjusted for questionnaire and HIV-testing non-response. In addition, analyses of response rates from the 2012 survey found that missingness had little impact on the HIV prevalence estimates by socio-demographic characteristics [21]. Besides limitations the strength of this study is that survey data are based on a large nationally representative sample that can be generalised to the South African population.

## Conclusions

The observed patterns of age difference between sexual partners and associated factors are indirect indicators of economics and power differentials between the different sexes. The findings provide evidence that age-disparate relationships are an important contributor to the continued high transmission of HIV especially among adolescent girls and young women in the country. There is a need to strengthen interventions aimed at increasing female education including power to negotiate safer sex and economic independence combined with behaviour change interventions to address risky sexual behaviours and unprotected sex. At the same time there is a need to engage men to address harmful gender norms.

## Data Availability

The dataset(s) are available through the Human Sciences Research Council data research repository via access dataset http://www.hsrc.ac.za/en/research-data/uponrequest.

## Abbreviations

AUDIT: Alcohol Use Disorder Identification Test
CI: Confidence intervals
CDC: Centers for Disease Control and Prevention
EAs: Enumeration areas
HIV: Human immunodeficiency virus
RRR: Relative risk ratio
SES: Socio-economic status
VP: Visiting points
